# Genomic analysis of the relationship between gene expression variation and DNA polymorphism in *Drosophila simulans*

**DOI:** 10.1186/gb-2008-9-8-r125

**Published:** 2008-08-12

**Authors:** Mara KN Lawniczak, Alisha K Holloway, David J Begun, Corbin D Jones

**Affiliations:** 1 Division of Cell and Molecular Biology, Imperial College London, London, SW7 2AZ, UK; 2 Department of Evolution and Ecology and Center for Population Biology, University of California, Shields Avenue, Davis, CA 95616, USA; 3 Department of Biology and Carolina Center for Genome Science, University of North Carolina, Chapel Hill, NC 27599, USA

## Abstract

**Background:**

Understanding how DNA sequence polymorphism relates to variation in gene expression is essential to connecting genotypic differences with phenotypic differences among individuals. Addressing this question requires linking population genomic data with gene expression variation.

**Results:**

Using whole genome expression data and recent light shotgun genome sequencing of six *Drosophila simulans *genotypes, we assessed the relationship between expression variation in males and females and nucleotide polymorphism across thousands of loci. By examining sequence polymorphism in gene features, such as untranslated regions and introns, we find that genes showing greater variation in gene expression between genotypes also have higher levels of sequence polymorphism in many gene features. Accordingly, X-linked genes, which have lower sequence polymorphism levels than autosomal genes, also show less expression variation than autosomal genes. We also find that sex-specifically expressed genes show higher local levels of polymorphism and divergence than both sex-biased and unbiased genes, and that they appear to have simpler regulatory regions.

**Conclusion:**

The gene-feature-based analyses and the X-to-autosome comparisons suggest that sequence polymorphism in *cis*-acting elements is an important determinant of expression variation. However, this relationship varies among the different categories of sex-biased expression, and *trans *factors might contribute more to male-specific gene expression than *cis *effects. Our analysis of sex-specific gene expression also shows that female-specific genes have been overlooked in analyses that only point to male-biased genes as having unusual patterns of evolution and that studies of sexually dimorphic traits need to recognize that the relationship between genetic and expression variation at these traits is different from the genome as a whole.

## Abstract 2

Analysis of six *Drosophila simulans* genotypes revealed that genes with greater variation in gene expression between genotypes also have higher levels of sequence polymorphism in many gene features.

## Background

Phenotypic differences among individuals result, in part, from variation in gene expression caused by underlying sequence polymorphism. Thus, a deeper understanding of the relationship between sequence polymorphism and expression variation (defined here as within species differences in transcript abundance across genotypes) is a crucial component of connecting genotype to phenotype and of elucidating the mechanisms of phenotypic evolution. Several previous studies have combined genome-wide gene expression data with divergence estimates in protein coding regions to investigate the relationship between genotype and phenotype. For example, genes that show significant expression variation within species tend to be more diverged at amino acid sites between species and are often male-biased in their expression [1-4]. The same patterns are found for genes that have diverged in expression between species [3,5-7]. Finally, more highly expressed genes tend to show lower levels of both polymorphism and divergence in coding regions [1,3,8].

Sequence variation of *cis*-acting regulatory regions is clearly important in determining expression differences within species [9,10] and between species [7,11,12] (reviewed in [13,14]). Several recent studies have also shown that expression variation within a species is correlated with local levels of nucleotide heterozygosity [8,15,16]. However, in many studies, expression variation could have been confounded with sequence variation, as there has been no way of evaluating or correcting for probe mismatch between the strains used and the reference upon which the expression array was designed. We examine expression variation in genotypes that have been recently whole-genome shotgun sequenced [17], which provides us with the information necessary to mask probes that show differences from the reference sequence. The genome sequence data also give us accurate estimates of nucleotide heterozygosity within gene features for the same genotypes, which allows us to investigate the connection between local sequence variation and expression variation on a genomic scale. Thus far, this relationship has been examined only in *Saccharomyces cerevisiae*, where an enrichment of sequence polymorphisms between two strains was observed in the promoter regions and the 3' untranslated regions (UTRs) of genes that showed expression differences between the strains [16].

A description of the genomic relationship between expression variation and local heterozygosity would allow one to begin investigating the connection between these sources of variation in different functional elements, such as UTRs, coding regions and introns, and provide some information regarding the physical scale over which sequence variation is correlated with expression variation. A strong positive correlation between nucleotide heterozygosity and expression variation would provide genomic evidence for the relationship between *cis*-acting sequence variants and expression variation. Furthermore, such a positive correlation would raise interesting questions about the population genetic factors influencing expression variation. Two population genetic models for explaining local variation in heterozygosity are hitchhiking effects of linked beneficial mutations and variation in neutral mutation rates. A positive correlation between heterozygosity and expression variation would suggest one of two mechanisms. First, recent hitchhiking events in *cis*-acting regions would reduce sequence variation and, therefore, expression variation. Under a second mechanism, if the neutral mutation rate were high, variation at *cis*-acting regulatory sites would be manifest as elevated variation in expression levels. Alternatively, a weak relationship between local levels of heterozygosity and expression variation might suggest that *trans*-acting effects are more important determinants of gene expression variability.

Here, we use whole genome polymorphism data to examine the relationship between sequence polymorphism and expression variation at a genomic scale. The strength of our data lies in having assessed gene expression variation from the same six *D. simulans *lines for which we have whole genome sequences. We also revisit the previously examined relationship of sequence divergence and gene expression variation using our *D. simulans *data in combination with the whole genome sequences of *Drosophila melanogaster *and *Drosophila yakuba*. Using these resources, we summarize sequence polymorphism and divergence in specific features of annotated genes including coding regions, UTRs, putative core promoter regions (CPRs), and introns. We then examine whether expression variation is related to sequence polymorphism (and divergence) in particular features at a genomic level.

A second focus of this work is to understand whether there are different relationships between expression variation and sequence polymorphism depending on chromosomal location, gene expression level, and sex biased expression. As there is clear evidence for reduced sequence polymorphism on the X chromosome [17], we ask whether there is reduced expression variation among X-linked genes compared to autosomal genes. Highly expressed genes have repeatedly been shown to be less polymorphic and evolve more slowly than lowly expressed genes [1,3,8] and we also examine whether these categories have different tendencies for variable expression. Finally, we examine the relationship between sequence polymorphism and expression variation for different categories of sex bias. As males and females share a common genome, sexual dimorphism is determined by differences in gene expression [18]. The factors controlling sexually dimorphic gene expression could be very different from those controlling unbiased gene expression. Comparison of sex-specific genes to unbiased genes will determine if the relationship between expression and genetic variation at sexually dimorphic genes is different from the genome as a whole.

## Results

### Gene expression variation and population genomic sequence data

Genome-wide summaries of sequence length, polymorphism and divergence for each gene feature for which we have detectable expression data are presented in Table 1. Our microarray data show 313 genes in males and 119 genes in females with significant expression variation between lines after Bonferroni correction. Taking a slightly less conservative approach (*p *< 0.001), 16% of genes (1,262/7,949) and 10% of genes (723/7,128 genes) show expression variation in males and females, respectively.

**Table 1:** Gene feature length, polymorphism and divergence by gene expression variation for each sex

		Male*				Female*			
			
	Genome average	NS^†^	SIG^‡^	*X*^2^	*p*-value^§^	NS^†^	SIG^‡^	*X*^2^	*p*-value^§^
Number of genes		6,687	1,262			6,405	723		
									
Length									
EXON	1,675	1,726	1,357	67.07	***	1,768	1,416	36.94	***
5'UTR	239	251	198	59.68	***	248	216	16.59	***
Intron	2,493	2,750	1,764	16.14	***	2,598	2,390	4.51	0.0336
Number of introns	3.55	3.69	3.11	16.42	***	3.67	3.11	13.68	0.0002
3'UTR	392	418	299	96.22	***	414	353	28.52	***
									
Polymorphism									
CPR	0.0290	0.0290	0.0284	0.88	0.3479	0.0297	0.0304	0.32	0.5727
5'UTR	0.0112	0.0108	0.0127	13.34	0.0003	0.0108	0.0122	5.94	0.0148
Nonsynonymous	0.0024	0.0022	0.0029	43.56	***	0.0021	0.0026	21.63	***
Synonymous	0.0318	0.0308	0.0357	62.93	***	0.0310	0.0355	28.04	***
First intron	0.0277	0.0274	0.0294	6.45	0.0100	0.0266	0.0284	6.82	0.0090
All introns	0.0302	0.0297	0.0324	12.53	0.0004	0.0290	0.0317	9.56	0.0020
3'UTR	0.0122	0.0114	0.0156	66.80	***	0.0110	0.0151	54.52	***
									
Divergence^¶^									
CPR	0.0525	0.0532	0.0468	26.96	***	0.0543	0.0514	3.16	0.0757
5'UTR	0.0229	0.0224	0.0225	0.01	0.9063	0.0223	0.0216	0.11	0.7392
Nonsynonymous	0.0060	0.0057	0.0065	17.96	***	0.0049	0.0054	13.64	0.0002
Synonymous	0.0531	0.0526	0.0538	5.41	0.0200	0.0522	0.0541	5.79	0.0160
First intron	0.0463	0.0457	0.0472	3.07	0.0797	0.0448	0.0480	3.70	0.0546
All introns	0.0487	0.0480	0.0503	4.98	0.0256	0.0472	0.0512	9.11	0.0025
3'UTR	0.0228	0.0217	0.0256	22.61	***	0.0209	0.0244	20.22	***

*Male and female sets include genes that are expressed in that sex, but may also be expressed in the other sex. ^†^NS, not significantly differentially expressed between genotypes (AOV *p*-value > 0.001). ^‡^SIG, significantly differentially expressed between genotypes (AOV *p*-value ≤ 0.001). ^§^*X*^2 ^and *p*-values derived from Kruskal Wallis; three asterisks denote *p*-value < 0.0001. ^¶^Divergence refers to lineage specific divergence along the *D. simulans *branch.

**Figure 1:**
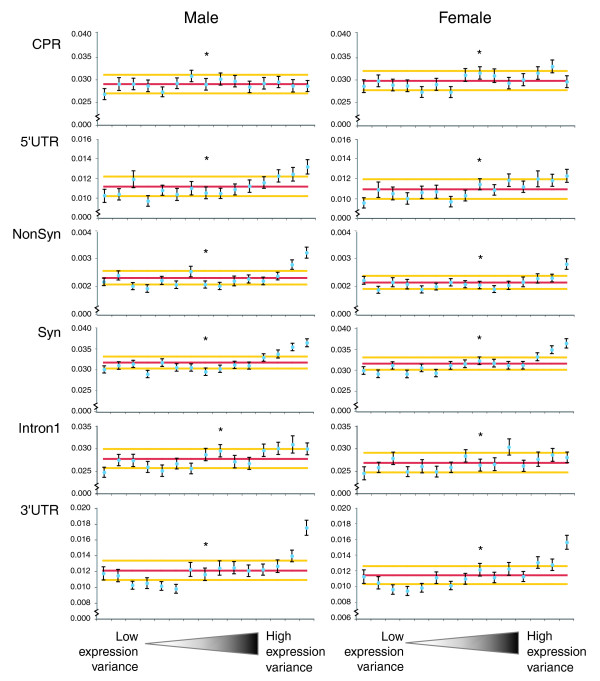
Significant expression variation between genotypes is associated with elevated levels of sequence polymorphism at most types of sites. The y-axis is the per site nucleotide diversity (note: axis scale varies by feature). The pink line indicates the genomic mean nucleotide diversity and yellow lines indicate 95% confidence intervals around the genomic mean. The x-axis represents the level of expression variation between genotypes for the different gene features as named (5'UTR, untranslated region; CPR, core promoter region; NonSyn, nonsynonymous sites; Syn, synonymous sites). *P*-values from the AOV of expression variation were sorted and grouped into 15 equal sized bins. Bins on the left side of the figure have no evidence of expression variation and bins on the right have the most variably expressed genes. For each bin, blue circles represent the mean nucleotide diversity with standard error bars. Permutation tests examined whether nucleotide diversity was higher within each bin than in a random sample of genes from the genome. The asterisk marks the bin in which an average *p*-value = 0.05 occurs. To the right of the asterisk, a positive trend is observed in some gene features, suggesting that the positive relationship between gene expression variation and nucleotide polymorphism is not solely confined to the most dramatically differentially expressed genes.

Variably expressed genes (*p *< 0.001) show significantly higher nucleotide heterozygosity in all gene features except for the putative 5' CPR (see Materials and methods for definition). This relationship extends beyond the genes exhibiting the most dramatic expression variation (Figure 1) and is visible even among genes that have marginal expression variation (*p *< 0.05, noted with asterisks in Figure 1). Figure 1 shows that the positive relationship between π and expression variation is strong for the coding regions and 3'UTRs, weak for introns and 5'UTRs, and is absent for CPRs. These results are robust to different bin sizes (Materials and methods). Variably expressed genes also have significantly shorter coding sequences, 5'UTRs, intronic regions, and 3'UTRs, and significantly fewer introns than non-variably expressed genes in both sexes (Table 1). In other words, variably expressed genes are shorter and more polymorphic than other genes.

We have done our best to remove the possibility that the relationship between expression variation and nucleotide heterozygosity is due to probe mismatch by removing all probes that show any divergence from the *D. melanogaster *sequence in addition to any polymorphism within the *D. simulans *genome sequences (see Materials and methods). However, due to the light coverage of the *D. simulans *genome sequences, for many probes we are missing sequence data for some genotypes. Therefore, we also exclude all probes that have fewer than two genotypes that show perfect concordance with the *D. melanogaster *probe sequence (coverage n ≥ 2). We also confirmed that our results were robust when we increased the stringency to n ≥ 4 at each site within a probe (Table S1 in Additional data file 1; see Materials and methods). Additionally, for any given gene, we found no significant difference in the average intensity (for example, expression level) between genotypes with no coverage in comparison to genotypes with sequence coverage (Materials and methods). Furthermore, for any given gene, the genotype that is most differentially expressed is missing sequence information no more frequently than expected by chance (χ^2 ^= 1.177, *p *= 0.2779). We repeated this analysis for the top 500 statistically significant genes and also found no effect. Finally, our results are robust even when we exclude all significantly differentially expressed genes for which the outlier genotype is missing sequence data (data not shown). These results strongly suggest that unobserved polymorphisms at probe sites are not confounding our analyses (see Materials and methods).

Similar to the relationship with polymorphism, expression variation in both sexes has a positive relationship with sequence divergence in coding regions, 3'UTRs and, to a lesser extent, introns (Table 1). However, the relationship between expression variation and heterozygosity is quite different from the relationship between expression variation and sequence divergence for some functional elements. For example, expression variation is positively associated with 5'UTR polymorphism, but not 5'UTR divergence (Table 1). Additionally, expression variation is significantly negatively associated with CPR divergence in the male analysis but shows no relationship with CPR polymorphism (Table 1).

### X-linkage

X-linked genes are far less likely than autosomal genes to vary between genotypes in expression, especially in males (Mann-Whitney U test (MWU): males *X*^2 ^= 55.25, *p *< 0.0001; females *X*^2 ^= 17.51, *p *< 0.0001). However, male-expressed X-linked genes have significantly lower average gene expression than autosomal genes (*X*^2 ^= 8.92, *p *= 0.0028) whereas female-expressed genes do not differ in their expression level depending on chromosomal location (*X*^2 ^= 0.06, *p *= 0.80). This lower gene expression intensity among male-expressed X-linked genes might reduce our ability to detect significant expression differences for this category. Even when we restrict our analysis to only average and highly expressed genes - thereby completely removing the significant difference in average gene expression intensity between X and autosomes - we find that the male-expressed X-linked genes are still less likely to show significant expression variation than are autosomal genes (*X*^2 ^= 35.25, *p *< 0.0001).

### Expression level

We find that most gene features of highly expressed genes are less heterozygous than those of average or lowly expressed genes (Tables 2 and 3 for males and females, respectively) yet highly expressed genes are more likely to show expression variation than average or lowly expressed genes as previously reported [1,3,8]. It is important to note that our reduced ability to detect expression variation in lowly expressed genes might contribute to the finding that highly expressed genes are more likely to show variable expression. Although highly expressed genes have lower overall levels of polymorphism, the positive relationships shown in Table 1 between sequence polymorphism in the various gene features and expression variation are still strong for average and highly expressed genes and weak for lowly expressed genes (data not shown). Highly expressed genes also show lower levels of divergence in UTRs, introns, and coding regions (Tables 2 and 3) consistent with previous reports [2,19,20]. However, the CPR shows the opposite trend, with highly expressed genes having greater heterozygosity and greater divergence (Tables 2 and 3). Highly expressed genes also tend to have shorter gene features and fewer introns than average expressed genes, which are, in turn, shorter than lowly expressed genes (Tables 2 and 3).

**Table 2:** Gene feature length, polymorphism and divergence in males for genes with high, average and low levels of expression

	Low	Average	High	Tukey's HSD summary*	*X*^2^	*p*-value^†^
Number of genes	2,073	4,167	1,709			
						
Length						
EXON	1,874	1,747	1,225	L>A>H	306.91	***
5'UTR	282	240	207	L>A>H	16.62	0.0002
Intron	3,644	2,521	1,450	L>A>H	7.11	0.0286
Number of introns	3.86	3.75	2.88	L=A>H	63.89	***
3'UTR	503	380	338	L>A>H	77.77	***
						
Polymorphism						
CPR	0.0277	0.0295	0.0290	L=A=H	13.40	0.0012
5'UTR	0.0114	0.0116	0.0097	L=A>H	24.01	***
Nonsynonymous	0.0029	0.0023	0.0016	L>A>H	245.83	***
Synonymous	0.0335	0.0322	0.0277	L>A>H	86.68	***
First intron	0.0290	0.0276	0.0263	L≥A≥H	7.58	0.0226
All introns	0.0317	0.0301	0.0284	L≥A≥H	9.52	0.0086
3'UTR	0.0131	0.0122	0.0109	L=A>H	41.23	***
						
Divergence^‡^						
CPR	0.0493	0.0533	0.0528	A=H>L	19.77	***
5'UTR	0.0225	0.0232	0.0208	A≥L≥H	12.66	0.0018
Nonsynonymous	0.0066	0.0060	0.0047	L>A>H	155.62	***
Synonymous	0.0524	0.0543	0.0494	A≥L≥H	35.69	***
First intron	0.0475	0.0463	0.0433	L=A>H	7.79	0.0203
All introns	0.0483	0.0492	0.0462	A≥L≥H	7.06	0.0293
3'UTR	0.0237	0.0225	0.0204	L=A>H	22.83	***

*L, low expression; A, average expression; H, high expression (see Materials and methods). ^†^*X*^2 ^and *p*-values derived from Kruskal Wallis; three asterisks denote *p*-value < 0.0001. ^‡^Divergence refers to lineage specific divergence along the *D. simulans *branch.

**Table 3:** Gene feature length, polymorphism and divergence in females for genes with high, average and low levels of expression

	Low	Average	High	Tukey's HSD summary*	*X*^2^	*p*-value^†^
Number of genes	1,652	3,999	1,477			
						
Length						
EXON	1,877	1,825	1,319	L=A>H	198.71	***
5'UTR	287	241	213	L>A>H	15.53	0.0004
Intron	3,845	2,521	1,386	L>A>H	20.64	***
Number of introns	3.97	3.71	2.94	L=A>H	48.06	***
3'UTR	547	366	384	L>H=A	108.03	***
						
Polymorphism						
CPR	0.0260	0.0304	0.0315	H=A>L	59.21	***
5'UTR	0.0110	0.0115	0.0094	A=L>H	28.06	***
Nonsynonymous	0.0028	0.0021	0.0013	L>A>H	341.18	***
Synonymous	0.0337	0.0325	0.0259	L=A>H	148.96	***
First intron	0.0283	0.0272	0.0240	L=A>H	19.94	***
All introns	0.0309	0.0298	0.0260	L=A>H	24.77	***
3'UTR	0.0136	0.0116	0.0089	L>A>H	106.22	***
						
Divergence^‡^						
CPR	0.0452	0.0550	0.0597	H>A>L	132.46	***
5'UTR	0.0218	0.0229	0.0209	A≥L≥H	6.70	0.0350
Nonsynonymous	0.0066	0.0048	0.0034	L>A>H	243.38	***
Synonymous	0.0513	0.0546	0.0476	A≥L≥H	79.80	***
First intron	0.0471	0.0459	0.0411	L=A>H	13.88	0.0010
All introns	0.0482	0.0489	0.0437	A=L>H	22.22	***
3'UTR	0.0241	0.0221	0.0168		74.98	***

*L, low expression; A, average expression; H, high expression (see Materials and methods). ^†^*X*^2 ^and *p*-values derived from Kruskal Wallis; three asterisks denote *p*-value < 0.0001. ^‡^Divergence refers to lineage specific divergence along the *D. simulans *branch.

### Sex bias

Genes were divided into five sex-related categories - male-specific, male-biased, female-specific, female-biased, and unbiased (see Materials and methods). The relationship between nucleotide variation, expression variation, and sex bias is complicated but several general patterns emerge (Table 4; see Table S2 in Additional data file 2 for more details). Polymorphism in coding regions and 5'UTRs is significantly higher in sex-specific genes than non-sex-specific genes (the pooled class of sex-biased and unbiased genes). Male-specific and male-biased genes have lower levels of polymorphism in the CPR than other genes, but higher levels of polymorphism in introns and 3'UTRs. Overall, sex-specific genes show greater levels of divergence in most gene features; however, rates of amino acid evolution in male-specific genes are strikingly higher than all other classes of bias (Table 4). In contrast, in the CPR, female-biased and female-specific genes are evolving more rapidly than unbiased genes, which are, in turn, evolving more rapidly than male-biased and male-specific genes (Table 4). Coding sequence length also shows a strong relationship with sex bias (Table 4). Female-specific and female-biased coding regions are longer than unbiased genes, which are, in turn, longer than male-biased and male-specific genes. Sex-specific genes have significantly shorter UTRs and significantly fewer introns than sex-biased and unbiased genes (Table 4). This result is somewhat surprising for female-specific genes as they have among the longest coding regions.

**Table 4:** Gene feature length, polymorphism and divergence for sex-specific*, sex-biased*, and unbiased genes

	*X*^2^	*p*-value^†^	Tukey's HSD summary^‡^	Summary^§^
Number of genes				
				
Length				
EXON	247.10	***	Fb≥Fs≥U≥Mb≥Ms	F>U>M
5'UTR	133.27	***	U,Fb≥Mb≥Ms,Fs	NSS>SS
Intron	131.81	***	U≥Mb,Fb,Ms>Fs	NSS>SS
Number of introns	64.44	***	U,Fb≥Mb≥Ms,Fs	NSS>SS
3'UTR	236.01	***	U≥Fb≥Mb>Ms,Fs	NSS>SS
5' intergenic	291.9	***	Ms>Mb,U,Fs≥Fb	M>F,U
3' intergenic	274.6	***	Ms≥Mb≥U,Fb,Fs	M>F,U
				
Polymorphism				
CPR	79.64	***	Fb,Fs,U>Ms,Mb	F,U>M
5'UTR	22.14	0.0002	Ms,Fs≥Fb,Mb≥U	SS>NSS
Nonsynonymous	305.11	***	Ms≥Fs≥Mb≥U,Fb	SS>NSS
Synonymous	33.62	***	Fs,Ms≥Mb≥U,Fb	SS>NSS
First intron	59.49	***	Ms≥Mb≥Fs,U,Fb	M>F,U
All introns	48.10	***	Ms≥Mb≥Fs,Fb,U	M>F,U
3'UTR	156.48	***	Ms≥Mb≥Fs>U>Fb	M>F,U
				
Divergence^¶^				
CPR	212.79	***	Fb,Fs>U>Ms,Mb	F>U>M
5'UTR	80.02	***	Fs≥Ms>Fb≥Mb≥U	SS>NSS
Nonsynonymous	533.92	***	Ms>Fs,Mb>Fb,U	SS>NSS
Synonymous	81.82	***	Ms≥Fs,Fb,Mb≥U	SS>NSS
First intron	68.47	***	Ms,Fs≥Mb≥Fb,U	SS>NSS
All introns	55.72	***	Ms≥Fs≥Mb≥Fb,U	SS>NSS
3'UTR	259.87	***	Ms≥Fs≥Mb≥Fb≥U	SS>NSS

*Male- and female-specific sets include genes that are expressed only in that sex, whereas sex-biased are expressed, on average, three-fold higher in one sex than the other. ^†^*X*^2 ^and *p*-values derived from Kruskal Wallis; three asterisks denote *p*-value < 0.0001. ^‡^Ms, male-specific; Mb, male-biased; Fs, female-specific; Fb, female-biased; U, unbiased. ^§^F, female; M, male; U, unbiased; NSS, non-sex-specific; SS, sex-specific. ^¶^Divergence refers to lineage specific divergence along the *D. simulans *branch.

## Discussion

### Gene expression variation and population genomic sequence data

The recent analysis of six genomes of *D. simulans *provided the first glimpse of whole genome population variation in a higher eukaryote [17]. We used polymorphism and divergence estimates for gene features (for example, UTRs, introns, and so on) together with expression variation measured using Affymetrix gene expression arrays (see Materials and methods) to examine the relationship between expression variation and local sequence polymorphism. Local or *cis *variation can affect gene transcription by modifying enhancer, promoter, or microRNA (miRNA) target sites. However, local sequence variation can also mislead us with respect to gene expression variation if probes hybridize differently due to undetected sequence polymorphism. Recent findings suggesting that protein divergence between species strongly correlates with expression divergence between species (for example, [2,3]) have been called into question [21]. Larracuente *et al*. [21] examined expression and protein divergence for seven *Drosophila *species using species-specific arrays. They found that expression divergence is largely uncoupled from protein divergence and they suggest that hybridization mismatch errors might have confounded previous research. Although we only examine gene expression variation within a species here, it is important to point out that the probe sequence issues are similar and can bias our results as polymorphism in probe regions can also cause errors in our measurements of transcription. We ameliorated this problem by: first masking probes that showed any divergence from *D. melanogaster *(on which the chip was based) or any polymorphisms within *D. simulans*; second, examining whether our results are robust to different coverage stringencies when there are missing data (they are); and third, examining whether genotypes with missing probe sequence data are more likely to be expression outliers than expected by chance (they are not). After these corrections and tests, we found a positive relationship between nucleotide polymorphism and expression variation that is particularly strong for coding regions and 3'UTRs (Table 1, Figure 1). While the strong positive relationship between nucleotide polymorphism and expression variation observed for features of the transcript suggests that the physical scale over which heterozygosity is correlated with expression variation may be gene-sized or larger, the results also suggest that smaller scale effects of heterozygosity may occur, as the relationship is quite different for the 3'UTR versus the core promoter region.

#### 3'UTR evolution

This first demonstration of a genome-wide positive relationship between expression variation and nucleotide polymorphism in the 3'UTR suggests a functional link between these types of variation. 3'UTRs contain several types of regulatory elements, including binding sites for miRNAs and AU-rich elements, which are known to regulate gene expression. For example, miRNAs can bind and control protein abundance by suppressing translation or marking mRNAs for degradation (reviewed in [22]). In animals, knockouts of miRNAs produce variable results, ranging from no observable phenotype to developmental-stage specific death [23]. This indicates that, in many cases, miRNA-based regulation is both redundant with other methods of control and could be more important in fine-tuning protein levels rather than causing dramatic changes in abundance [23]. Also, analyses examining gene expression divergence across species in known miRNA target genes find that these genes are less likely to show expression divergence than non-targets [24]. Given these results, it is unclear whether there would be broad scale patterns observable between expression variation and sequence polymorphism in miRNA target genes. Nevertheless, miRNAs are thought to have a large impact on 3'UTR evolution with selection limiting miRNA complementary sites and 3'UTR length (thus avoiding additional binding sites) [25]. These patterns all suggest that the expression variation we observe to be tightly correlated with 3'UTR variation is unlikely to be caused by miRNA regulation. To further explore this, we examined the set of all predicted target miRNA targets [26] (retrieved from [27]) and we find that polymorphism in the 3'UTR of target genes is dramatically lower than non-targets (target 3'UTR average π = 0.00795 (n = 2,945); non-target 3'UTR average π = 0.0147 (n = 5,526); *X*^2 ^= 185.28, *p *< 0.0001). Of course, this is perhaps not surprising given that targets were identified by conservation in binding sites across many *Drosophila *species, and thus are likely highly conserved functionally [26]. However, the relationship between 3'UTR variation and expression variation among genes with known miRNA targets is also much weaker (target 3'UTR π in SIG (significantly varying genes) = 0.0087, NS (non-significantly varying genes) = 0.0077, *X*^2 ^= 6.21, *p *= 0.0127; non-target 3'UTR π in SIG = 0.0185, NS = 0.0138, *X*^2 ^= 49.04, *p *< 0.0001). This might further suggest that miRNA target site polymorphism is not a major contributor to expression variation, although it is important to note that our power to detect the relationship is also reduced, given lower levels of 3'UTR polymorphism.

Interestingly, a recent study reported that adaptive evolution of the 3' regulatory sequence is associated with recently evolved increased levels of expression in *D. simulans *[6]. Our results provide further support that the functional elements in the 3'UTR harbor sequence variants with significant impacts on expression variation. Although expression variation within species may not be related to miRNA control, there are many other aspects of the 3'UTR that can affect transcript abundance [28-30].

#### Core promoter region evolution

Unlike all other gene features examined here, heterozygosity in the CPR shows no strong evidence of a link with expression variation (Table 1, Figure 1). This is somewhat surprising as CPRs presumably include regulatory elements that might contain polymorphisms that contribute to expression variation. A recent study examining polymorphism in the upstream 1-2 Kb of a small set of genes that vary and do not vary in expression between *D. melanogaster *genotypes also found no relationship between upstream polymorphism and gene expression differences [31]. We suggest several possible explanations for this result. First, while the CPR might be functionally important for gene regulation, polymorphism at a small number of sites may be responsible for expression variation, thus preventing us from detecting a genomic relationship. Alternatively, CPR variants affecting expression variation may occur at low frequency and make only a small contribution to heterozygosity. For either of these two scenarios to be true, one must assume that CPR variants evolve under a distinctly different evolutionary regime than other types of either coding or non-coding variation. We have no evidence for this unusual assumption. In fact, our comparisons between the X and the autosomes show that levels of expression variation reflect overall patterns of sequence variation, suggesting the action of common evolutionary mechanisms. Thus, our first two explanations seem implausible. Instead we suspect that heterozygosity in *trans*-acting factors that interact with CPRs may instead shape the CPR's role in expression variation, perhaps leading to constraint in this region. From a population genetics perspective, however, we would expect to see reduced heterozygosity in CPRs relative to other gene features if they have greater functional constraint and this general pattern was not observed; in fact, UTRs are much less polymorphic and diverged than CPRs (Table 1).

However, if genes are examined by sex bias, this relationship changes. Male-biased and male-specific genes show significantly lower levels of polymorphism and divergence in the CPR than other categories of bias (Table 4). Furthermore, in spite of showing no relationship with heterozygosity in the CPR, variably expressed genes in males show reduced levels of divergence in the CPR (Table 1; Figure S1 in Additional data file 3). This is not true for variably expressed genes in females. Sequence conservation in the CPR among genes that are variably expressed in males supports the idea that the CPRs of these genes experience functional constraint because they contain important regulatory elements. This is the case for TATA-box containing genes, which are more variably expressed than TATA-less genes. TATA-box containing genes have twice as many transcription factor binding sites on average than TATA-less genes and thus show higher levels of sequence conservation in the CPR [32]. We find this pattern in our data, too, with TATA-box containing genes having much lower levels of polymorphism and divergence in the CPR, yet being significantly more likely to show expression variation (data not shown). Furthermore, TATA-box containing genes show no relationship between expression variation and nucleotide variation for any of the gene features. TATA-box containing genes, therefore, might be more likely to be influenced by distant *cis *or by *trans*-acting variation than local *cis *variation. In a recent study, a mutated TATA-box was demonstrated to have less frequent and lower magnitude transcriptional bursts than a conserved TATA-box, suggesting that the conserved TATA-box facilitates the formation of a stable transcription scaffold and this allows for rapid bursts of transcription [33]. Indeed, TATA-box containing genes are more likely to be stress-response genes, which must be capable of rapid bursts of transcription. In *Arabidopsis*, genes observed to change regulation under a variety of conditions (multi-stimuli response genes) have a greater likelihood of containing a TATA-box, a higher density of *cis*-elements in upstream regions, and longer upstream intergenic regions [34]. These multi-stimuli response genes are also shorter and have fewer introns so might be produced more economically [34]. Interestingly, all the patterns mentioned above for TATA-box containing genes are also true for male-biased genes; they tend to be more variably expressed, shorter, contain fewer introns and they have higher levels of conservation in the CPR. Furthermore, male-specific and male-biased genes show much greater upstream and downstream intergenic distances (Table 4), again similar to TATA-box containing genes. Perhaps male-specific and male-biased genes are more likely to be under the control of distant *cis*-regulatory elements or *trans*-factors. This could allow for the decoupling of local *cis *variation affecting expression from coding sequence variation. If the mutational target for expression changes is farther away from the coding sequence, then each can evolve more independently of the other. Male-biased and male-specific genes are notoriously rapidly evolving and a mechanism that decouples this rapid evolution from linked expression changes and allows each phenotype to evolve independently of the other could be beneficial. In a mutation accumulation experiment in yeast, the *trans *mutational target size and the presence of a TATA-box were each positively correlated with the likelihood that a gene changed in expression over time [35]. Male-biased gene expression is very labile over time [36], perhaps suggesting again that these genes are more influenced by *trans *variation than *cis *variation.

### X-linkage

Our results support previous research showing that the X chromosome is depleted of male-biased and male-specific genes and enriched for female-biased and female-specific genes (Table 4) [5,37,38]. A novel finding in our analyses is that the lower sequence polymorphism often observed on the X chromosome is reflected in less variable expression of X-linked genes, especially in males. This relationship supports the finding that local sequence variation and expression variation are linked. We find that males also have significantly lower average gene expression on the X than autosomes. The chromosome biology of the X and autosomes differs greatly as males are hemizygous for the X. In a majority of X-linked genes, dosage is equalized through hypertranscription mediated by the dosage compensation complex [39]. Incomplete dosage compensation on the X in males is a possible source of reduced average expression [39]. However, even after removing lowly expressed genes, males have significantly fewer variably expressed X-linked genes than autosomal genes.

### Expression level

Consistent with previous research, genes expressed highly in both sexes are more likely to show significant expression variation than average or lowly expressed genes (*X*^2 ^= 56.96, *p *< 0.0001; [2]), but, as noted, this may be due to technical difficulties in detecting differences in expression of lowly expressed genes. Highly expressed genes also tend towards lower levels of sequence polymorphism and divergence in UTRs, introns, and coding regions (Tables 2 and 3). These results extend and support findings from previous work that showed coding regions of highly expressed genes evolve slowly [2,19]. However, the CPR does not follow this pattern. In females, lowly expressed genes actually have lower levels of polymorphism in the CPR than average or highly expressed genes (Tables 2 and 3). Furthermore, this is the only category that shows a relationship where CPR polymorphism is positively associated with gene expression variation. This result may reflect the fact that, in the female analysis, there is an excess of male-biased genes in the lowly expressed class and male-biased genes tend to have particularly low levels of polymorphism in the CPR. Divergence in the CPR also shows a departure from patterns detected in the other gene features. Lowly expressed genes show lower levels of divergence in the CPR (Tables 2 and 3). This may be driven by a difference in the sexes discussed below.

### Sex bias

#### Sex-specific genes are highly polymorphic and evolve rapidly

Our study reveals that both female-specific and male-specific genes show elevated levels of polymorphism in coding regions and 5'UTRs while female-biased and male-biased genes show patterns more similar to unbiased genes (Table 4). Sex-specifically expressed genes also show elevated levels of divergence in all gene features except the CPR (Table 4). Indeed, the pooling of sex-specific and sex-biased genes in previous work might have masked the difference between these very different categories of expression.

The CPR stands out among the gene features because it shows the lowest levels of polymorphism and divergence among male-specific and male-biased genes in spite of the fact these genes show among the highest levels of polymorphism and divergence in all other gene features. It has been previously reported that male-biased genes are overrepresented among the class of genes that show expression variation [4] and divergence [36]. As discussed above, we speculate that there might be a difference between the locations of regulatory regions of male-biased versus female-biased and unbiased genes.

#### Sex-specific genes have simpler regulatory regions

Genes expressed in a sex-specific manner may have a more narrowly defined function than genes expressed in both sexes. Our data support this idea if the information content of UTRs and introns is correlated with their length and/or conservation. As previously mentioned, sex-specific genes show the highest levels of polymorphism and divergence in the UTRs and introns. Additionally, sex-specific genes have significantly shorter UTRs and significantly fewer introns than sex-biased and unbiased genes (Table 4). In fact, female-specific genes have the shortest UTRs and introns even though they have among the longest coding regions. The shorter introns and UTR suggests that there is less opportunity for information content in UTRs and introns in sex-specific genes.

To explicitly test the hypothesis that UTRs of sex-specific genes have fewer regulatory elements, we examined the 5'UTRs of sex-specific (SS) and unbiased genes (non-sex specific (NSS)) for evidence of translational regulatory elements. One mechanism of translational regulation is through upstream translation initiation codons (uAUGs) and upstream open reading frames (uORFs). These uAUGs and uORFs reside in the 5'UTR and can regulate translation by causing the ribosome to stall or by blocking another ribosome from the translation start site (see [40,41] for reviews). Based on the probability of observing an AUG given the base composition of the 5'UTR sequence, non-conserved AUGs are under-represented in 5'UTRs [40,41]. However, uAUGs conserved between species are overrepresented, which suggests that they serve some functional role.

We investigated the prevalence of conserved uAUGs and uORFs (present in *D. simulans*, *D. melanogaster*, and *D. yakuba*) in sex-specific and unbiased genes with 5'UTRs that were at least 50 nucleotides in length. For our analyses, uORFs are defined as having both an initiation and termination codon within the 5'UTR, whereas uAUGs are simply initiation codons in the 5'UTR that may or may not be followed by a termination codon. We find that sex-specific genes have fewer uORFs per 5'UTR nucleotide (MWU: SS 0.0036 versus NSS 0.0039, *X*^2 ^= 7.49, *p *= 0.0062) and a lower proportion of genes with a uORF present (MWU: SS 0.44 versus NSS 0.51, *X*^2 ^= 16.54, *p *< 0.0001). However, the pattern with uAUGs was less clear. There were similar numbers of uAUGs (per 5'UTR nucleotide) in sex-specific and non-sex-specific genes (MWU: SS 0.0051 versus NSS 0.0047, *X*^2 ^= 0.33, *p *= 0.5677), but there was a trend towards a lower proportion of sex-specific genes harboring uAUGs (MWU: SS 0.52 versus NSS 0.55, *X*^2 ^= 3.08, *p *= 0.0793). Given these data, we have weak evidence that sex-specific genes have fewer translational regulatory elements in their 5'UTRs, supporting the hypothesis that genes with more narrowly defined functions have simpler or fewer regulatory sequences.

## Conclusion

Across six genotypes of *D. simulans*, we find that genes with significant expression variation also tend to have higher levels of sequence polymorphism, particularly in the coding region and 3'UTR (Table 1, Figure 1). Clearly, *cis*-regulatory variation plays an important role in determining transcript levels, but these data cannot address the relative role of *trans*-acting factors. Further research examining the role of the 3'UTR in *Drosophila *gene expression will determine whether the positive association detected here indicates functional differences that may be acted upon by natural selection. Additional support for the positive relationship between sequence polymorphism and gene expression variation comes from comparisons of the X to autosomes. Genes located on the X, already known to have lower levels of sequence polymorphism than autosomal genes [42], are also less likely to show significant expression variation than genes on autosomes. Similar to previously published reports, we find that sex differences in expression are abundant and male-biased genes are overrepresented among the most variably expressed genes [4]. However, by pooling sex-specific genes with sex-biased genes, some information is lost. We find that female-specific genes are a previously overlooked category showing high levels of polymorphism and divergence for some gene features. Additionally, these sex-specific genes may have simpler mechanisms of gene regulation related to fewer or more narrowly defined functions. This last point has important implications for studies examining the importance of regulatory changes in the evolution of phenotypic differences as it implies that patterns inferred from sexually dimorphic traits might not be reflective of the genome as a whole.

## Materials and methods

### Genotypes

Gene expression and sequence data are derived from seven genotypes of *D. simulans *from Kenya (*c167.4*), Madagascar (*md106 *and *md199*), New Caledonia (*nc48*), and North America (*w*^*501*^, *sim4*, and *sim6*). These genotypes were recently sequenced using light shotgun whole genome sequencing [17]. The inbred lines *sim4 *and *sim6 *are from a single population (Winters, California). Sequence reads of *sim4 *and *sim6 *were combined to produce a consensus genomic sequence [17].

### Sample preparation for microarray analysis

Parental flies from each genotype were reared on standard laboratory medium at room temperature in the same facility. Virgin flies from each of the seven genotypes were collected and housed in single sex vials in groups of ten for three days. On the morning of the fourth day, 3 replicates of 30 flies from each sex and each genotype were flash frozen. Flies were stored at -80°C until RNA extraction.

Total RNA was extracted from whole flies using Trizol reagent (Invitrogen, Carlsbad, CA, USA). Affymetrix guidelines were followed for cDNA synthesis, cRNA processing and biotin-labeling, and fragmenting. We analyzed gene expression variation in the seven genotypes discussed above using Affymetrix Dros2 *D. melanogaster *genechips. Oligonucleotide chips were probed, hybridized, stained, washed and scanned at the UC-Davis Core Facility according to Affymetrix guidelines.

### Microarray probe masking

The Dros2 Affymetrix chip has approximately 18,700 probesets, each representing a known or predicted transcript. Each probeset is composed of fourteen 25-base oligonucleotide probes that perfectly match (PM) the *D. melanogaster *reference sequence and 14 probes that mis-match (MM) the reference sequence at the central (13th) base of the probe. For our purposes, all data from the MM probes were excluded; thus, each probeset is represented by up to 14 PM probes.

Probes from the Affymetrix Dros2 genechips were developed from within target sequences of transcribed DNA. These target sequences correspond to transcribed sequence that may or may not be contiguous (that is, targets may span an intron, but do not include intronic sequence). Probes for the Affymetrix Dros2 genechips were designed from *D. melanogaster *assembly version 3 whereas our analyses are all based on assembly version 4. In order to reconcile two assembly versions and associate probesets with genes, we downloaded target sequences from Affymetrix [43] and identified homologous sequence in version 4 using BLAT [44]. We removed target sequences (and therefore probesets) that hit multiple locations within the *D. melanogaster *genome. We also removed probes that hit multiple locations within target sequences.

Using the light shotgun whole genome sequences available for the *D. simulans *genotypes assayed for gene expression, probe polymorphism within *D. simulans *and divergence from *D. melanogaster *was corrected for in our analyses. We followed the approach described in [45]. This and other earlier work noted that the effect of this divergence can be either a reduction of signal or an inflation of the variance in the signal among probes [45]. The latter, which can be an issue on Affymetrix arrays, tends to reduce the power of an analysis. As this should make it harder to see a significant association, our analyses should be conservative.

On average, in the syntenic assemblies there are sequence data from 3.9 *D. simulans *lines covering each nucleotide. For detection of polymorphism, we required that each nucleotide within a probe be represented by data from at least two *D. simulans *lines. Probes with lower coverage were masked, as were all probes that were found to be polymorphic within the six *D. simulans *genotypes. We also masked all probes that showed any sequence divergence between the *D. simulans *and *D. melanogaster *genome sequences. It is important to note that although including divergent probes might give inaccurate information as to true levels of gene expression, because we are only comparing gene expression within *D. simulans*, including divergent probes should not bias our results. The real concern for masking is the polymorphic probes within *D. simulans*. However, it should be noted that this has been considered but not been corrected for in intraspecific expression analyses in other species because it has been demonstrated to have a minor contribution to expression variation [3,4,8]. Regardless, it is possible that by considering probes that were covered by only two *D. simulans *lines, we missed some probes that were polymorphic within *D. simulans*. Therefore, we also conducted all analyses with a second dataset that included only probes that were covered by at least four *D. simulans *lines. The results are quantitatively identical to the analyses presented in the paper (Table S1 in Additional data file 1). As an additional approach towards determining whether missing sequence data could bias our results, we also examined whether the most significant outlier genotype in terms of gene expression was also one of the genotypes that was missing sequence data in the n ≥ 4 coverage analyses. If true - that is, if the missing genotypes for any given gene also tend to be expression outliers for that gene - then the missing probe sequence data could be different from the reference probe and thus have an impact on gene expression that is unrelated to actual expression. We limited our analysis to male data as these showed the most deviant expression. The most differentially expressed genotype was missing sequence data (n = 1,926) no more likely than expected by chance (n = 1,965; χ^2 ^= 1.177, *p *= 0.2779). Nor was there significant difference in the average intensity value between those genes with coverage and those without (genes with coverage, intensity = 6.882; genes without coverage, intensity = 6.865). This analysis, however, may be confounded by the fact that most genes are not statistically significantly different among lines and that inclusion of these genes in the analysis may obscure an effect. Thus, we repeated the analysis with the 500 genes with the strongest differences in expression among the lines. The expression outlier was missing sequence data in 161 cases, which is not significantly different from the random expectation of 167 (χ^2 ^= 0.324; *p *= 0.5694). Therefore, missing probe sequence data do not appear to bias our results.

In addition to masking divergent and polymorphic probes, we also masked probes that were called 'absent' by the Affymetrix algorithm. We did this to ensure that only genes with reliably detectable sequence were used in the analysis. Any probeset not called 'present' in at least three chips was considered to be absent (absent male = 7,817 probesets; absent female = 9,238 probesets). Masking was performed prior to background correction and normalization using code graciously sent by Ariel Chernomoritz. This was done for each sex separately as some genes are considered absent in one sex but present in the other.

### Microarray background correction, normalization, and analysis

After masking, data were processed and normalized using the *rma *package in Bioconductor [46]. Expression variation was assessed separately for each sex using mixed model ANOVAs in R with genotype as a fixed effect and replicate (RNA prep) as a random effect. The total number of genes analyzed after the removal of polymorphic and diverged probes and absent probesets was 7,949 for males and 7,128 for females.

### Estimates of nucleotide polymorphism and divergence

*D. simulans *and *D. yakuba *were syntenically aligned to v4 of the *D. melanogaster *genome assembly [17]. Genes in *D. simulans *and *D. yakuba *were assessed for initiation codons, splice junctions, and termination codons that matched the *D. melanogaster *gene model (annotation v4.2). Estimates of polymorphism, π, and divergence were taken from Begun *et al*. [17]. Briefly, the six *D. simulans *lines were used to estimate levels of nucleotide variation. In coding regions, π was calculated according to Nei and Gojobori [47] to count the number of nonsynonymous and synonymous sites and to determine the number of nonsynonymous and synonymous changes between two codons. Male flies are hemizygous for the X chromosome. Assuming there are equal numbers of males and females in a population, differences in population size between the X and autosomes were corrected for by multiplying polymorphism estimates on the X by 4/3. Lineage-specific divergence in *D. simulans *was estimated using *D. melanogaster *and *D. yakuba *reference sequences. In coding regions, divergence was calculated using codeml with codon frequencies estimated from the data and dN and dS estimated for each branch [48]. For noncoding regions, baseml [48] with HKY as the model of evolution was used to account for base frequency and transition/transversion bias [49].

Polymorphism data were summarized for the following gene features on a gene-by-gene basis. Three hundred bases just upstream of the transcription initiation site were examined because this region typically contains the core promoter (CPR). Predicted and gold collection (that is, those with a fully sequenced cDNA; retrieved from [50]) 5' and 3'UTRs were examined. We include analyses using the pooled set of predicted and gold UTRs because analyses using the more conservative gold UTR datasets did not differ from the pooled datasets. Both synonymous and nonsynonymous sites were examined for the coding regions of each gene. Additionally, because regulatory elements are often found in first introns, levels of polymorphism in the first intron and the combined data for all introns were examined separately. Divergence along the *D. simulans *lineage was also calculated for each of these features.

### Association of gene expression variation with population genomic data

The *p*-values resulting from the gene-by-gene ANOVAs were used to represent gene expression variability across genotypes, with low *p*-values indicating high levels of expression variation. Summary sequence data on polymorphism and divergence for each gene feature were combined with the ANOVA *p*-values. For each feature, genes were sorted by *p*-value and put into 15 equal sized bins with *n *genes in each bin. The average and standard error of π were calculated for each bin. For permutation tests, *n *π values were drawn from the total dataset and the average π was calculated. This was repeated 10,000 times to generate an empirical distribution of average π. The number of permuted datasets with average π values that were higher than the sample dataset (that is, each bin) was divided by the total number of permuted datasets to obtain *p*-values. This process was similar for investigation of the relationship between sequence divergence and expression variation.

In addition to discovering genes that vary in expression by genotype, we also categorized genes based on gene feature length and levels of expression intensity. Expression intensity was determined based on overall average expression for a gene on the chip. In males, the average gene expression intensity was 6.87 and in females it was 7.45. High, average, and low gene expression was determined by making cutoffs (males: low is less than 5.37, high is greater than 8.37; females: low is less than 5.95, high is greater than 8.95). These cut-offs are arbitrary and chosen because they resulted in about half of the genes falling into 'average' gene expression and the remainder of the genes falling roughly equally into 'high' and 'low' expression categories.

Classes of sex bias were determined by using both presence/absence calls and relative levels of gene expression. Genes were considered male-specific if they were called 'absent' in all female chips, but 'present' in at least three male chips. Female-specific genes were determined the same way. 'Strict' sex-specific genes were required to be present in all chips of one sex and absent in all chips of the other sex. Genes were considered male-biased if they were at least three-fold higher in males than females. Female-biased genes were determined the same way, and unbiased genes include all genes with less than three-fold variation in expression intensity between males and females.

## Abbreviations

CPR, core promoter region; miRNA, microRNA; MM, mismatch; MWU, Mann-Whitney U test; NSS, non-sex specific; PM, perfect match; SS, sex-specific; uAUGs, upstream translation initiation codons; uORFs, upstream open reading frames; UTR, untranslated region.

## Authors' contributions

ML helped design the experiments, collected and analyzed data, and wrote the paper. AH analyzed data and wrote the paper. DB helped design experiments and edited the paper. CJ helped design experiments, analyzed data and edited the paper.

## Additional data files

The following additional data are available. Additional data file 1 contains Table S1, which shows that the results presented in Table 1 and Figure 1 are robust to increasing the minimum coverage to four sequences per probe. Additional data file 2 contains Table S2, which shows the details of the statistical results summarized in Table 4. Additional data file 3 contains Figure S1, which shows the relationship between expression variation and divergence for the gene features discussed in this manuscript.

## Supplementary Material

Additional data file 1 The results presented in Table 1 and Figure 1 are robust to increasing the minimum coverage to four sequences per probe. Click here for file

Additional data file 2 Details of the statistical results summarized in Table 4. Click here for file

Additional data file 3 The relationship between expression variation and divergence for the gene features discussed in this manuscript. Click here for file
