# Supplementary material for: Genomic analysis of the relationship between gene expression variation and DNA polymorphism in Drosophila simulans
Source: Genome Biol. 2008 Aug 12;9(8):R125. doi: 10.1186/gb-2008-9-8-r125 (PMC2575515; doi:10.1186/gb-2008-9-8-r125)
Supplement: Additional data file 1 — The results presented in Table 1 and Figure 1 are robust to increasing the minimum coverage to four sequences per probe. [file gb-2008-9-8-r125-S1.doc]

Table S1

| Feature | Average π | Average *p*-value from expression analysis | Number in each bin | Low 95% CI | High 95% CI | *p*-value from permutation test |
| --- | --- | --- | --- | --- | --- | --- |
| CPR | 0.0267 | 0.8188 | 296 | 0.0272 | 0.0322 | 0.9796 |
|  | 0.0292 | 0.5670 |  |  |  | 0.6228 |
|  | 0.0305 | 0.4013 |  |  |  | 0.3052 |
|  | 0.0305 | 0.2868 |  |  |  | 0.2929 |
|  | 0.0287 | 0.2045 |  |  |  | 0.7513 |
|  | 0.0305 | 0.1385 |  |  |  | 0.3094 |
|  | 0.0306 | 0.0908 |  |  |  | 0.2823 |
|  | 0.0289 | 0.0572 |  |  |  | 0.6903 |
|  | 0.0300 | 0.0355 |  |  |  | 0.4320 |
|  | 0.0308 | 0.0202 |  |  |  | 0.2360 |
|  | 0.0294 | 0.0104 |  |  |  | 0.5814 |
|  | 0.0307 | 0.0047 |  |  |  | 0.2577 |
|  | 0.0291 | 0.0016 |  |  |  | 0.6484 |
|  | 0.0303 | 0.0003 |  |  |  | 0.3522 |
|  | 0.0300 | 1.13E-05 |  |  |  | 0.4205 |
| 5UTR | 0.0098 | 0.8157 | 289 | 0.0098 | 0.0121 | 0.9602 |
|  | 0.0101 | 0.5639 |  |  |  | 0.8864 |
|  | 0.0099 | 0.3989 |  |  |  | 0.9331 |
|  | 0.0096 | 0.2839 |  |  |  | 0.9777 |
|  | 0.0108 | 0.2009 |  |  |  | 0.5836 |
|  | 0.0110 | 0.1367 |  |  |  | 0.4744 |
|  | 0.0108 | 0.0898 |  |  |  | 0.5378 |
|  | 0.0102 | 0.0568 |  |  |  | 0.8592 |
|  | 0.0094 | 0.0352 |  |  |  | 0.9920 |
|  | 0.0109 | 0.0203 |  |  |  | 0.4968 |
|  | 0.0109 | 0.0105 |  |  |  | 0.4927 |
|  | 0.0113 | 0.0048 |  |  |  | 0.3056 |
|  | 0.0123 | 0.0016 |  |  |  | 0.0332 |
|  | 0.0134 | 0.0003 |  |  |  | 0.0002 |
|  | 0.0135 | 1.38E-05 |  |  |  | 0.0002 |
| Intron1 | 0.0268 | 0.8282 | 292 | 0.0251 | 0.0301 | 0.6870 |
|  | 0.0275 | 0.5785 |  |  |  | 0.4899 |
|  | 0.0255 | 0.4139 |  |  |  | 0.9101 |
|  | 0.0259 | 0.2949 |  |  |  | 0.8704 |
|  | 0.0259 | 0.2096 |  |  |  | 0.8652 |
|  | 0.0265 | 0.1403 |  |  |  | 0.7380 |
|  | 0.0256 | 0.0926 |  |  |  | 0.9010 |
|  | 0.0262 | 0.0590 |  |  |  | 0.8188 |
|  | 0.0265 | 0.0362 |  |  |  | 0.7412 |
|  | 0.0253 | 0.0207 |  |  |  | 0.9296 |
|  | 0.0289 | 0.0105 |  |  |  | 0.1849 |
|  | 0.0308 | 0.0047 |  |  |  | 0.0216 |
|  | 0.0309 | 0.0015 |  |  |  | 0.0192 |
|  | 0.0304 | 0.0003 |  |  |  | 0.0373 |
|  | 0.0306 | 1.12E-05 |  |  |  | 0.0282 |

| Table S1 (continued) | | |  |  |  |  |
| --- | --- | --- | --- | --- | --- | --- |
| Feature | Average π | Average *p*-value from expression analysis | Number in each bin | Low 95% CI | High 95% CI | *p*-value from permutation test |
| Nonsynonymous | 0.0021 | 0.8280 | 305 | 0.0019 | 0.0024 | 0.6840 |
|  | 0.0022 | 0.5699 |  |  |  | 0.4077 |
|  | 0.0019 | 0.4004 |  |  |  | 0.8962 |
|  | 0.0021 | 0.2871 |  |  |  | 0.6771 |
|  | 0.0019 | 0.2051 |  |  |  | 0.9236 |
|  | 0.0019 | 0.1393 |  |  |  | 0.8928 |
|  | 0.0021 | 0.0923 |  |  |  | 0.6077 |
|  | 0.0022 | 0.0590 |  |  |  | 0.3428 |
|  | 0.0018 | 0.0362 |  |  |  | 0.9821 |
|  | 0.0018 | 0.0204 |  |  |  | 0.9877 |
|  | 0.0021 | 0.0103 |  |  |  | 0.5215 |
|  | 0.0022 | 0.0044 |  |  |  | 0.3868 |
|  | 0.0024 | 0.0014 |  |  |  | 0.1023 |
|  | 0.0026 | 0.0003 |  |  |  | 0.0055 |
|  | 0.0029 | 9.54E-06 |  |  |  | <0.0001 |
| Synonymous | 0.0298 | 0.8280 | 305 | 0.0294 | 0.0327 | 0.8961 |
|  | 0.0299 | 0.5699 |  |  |  | 0.8725 |
|  | 0.0311 | 0.4004 |  |  |  | 0.4670 |
|  | 0.0276 | 0.2871 |  |  |  | 0.9998 |
|  | 0.0303 | 0.2051 |  |  |  | 0.7791 |
|  | 0.0306 | 0.1393 |  |  |  | 0.6677 |
|  | 0.0300 | 0.0923 |  |  |  | 0.8539 |
|  | 0.0297 | 0.0590 |  |  |  | 0.9154 |
|  | 0.0301 | 0.0362 |  |  |  | 0.8281 |
|  | 0.0295 | 0.0204 |  |  |  | 0.9417 |
|  | 0.0323 | 0.0103 |  |  |  | 0.1022 |
|  | 0.0324 | 0.0044 |  |  |  | 0.0899 |
|  | 0.0324 | 0.0014 |  |  |  | 0.0851 |
|  | 0.0344 | 0.0003 |  |  |  | 0.0002 |
|  | 0.0354 | 9.54E-06 |  |  |  | <0.0001 |
| 3UTR | 0.0107 | 0.8186 | 309 | 0.0101 | 0.0128 | 0.7866 |
|  | 0.0102 | 0.5610 |  |  |  | 0.9441 |
|  | 0.0096 | 0.3956 |  |  |  | 0.9911 |
|  | 0.0095 | 0.2814 |  |  |  | 0.9953 |
|  | 0.0096 | 0.1982 |  |  |  | 0.9936 |
|  | 0.0100 | 0.1356 |  |  |  | 0.9588 |
|  | 0.0117 | 0.0891 |  |  |  | 0.3291 |
|  | 0.0100 | 0.0566 |  |  |  | 0.9621 |
|  | 0.0119 | 0.0351 |  |  |  | 0.2651 |
|  | 0.0120 | 0.0203 |  |  |  | 0.2178 |
|  | 0.0127 | 0.0107 |  |  |  | 0.0560 |
|  | 0.0119 | 0.0048 |  |  |  | 0.2366 |
|  | 0.0120 | 0.0016 |  |  |  | 0.2272 |
|  | 0.0141 | 0.0003 |  |  |  | 0.0008 |
|  | 0.0146 | 1.09E-05 |  |  |  | 0.0001 |

^a^ Average level of polymorphism of elements in each bin.

^b^ Average *p*-values (from AOV of expression variation) of elements in each bin.

^c^ Number of elements in each bin.

^d^ 95% confidence intervals on polymorphism of elements in each bin.

^e^ *p*-values from 10000 permuted datasets.
