# Supplementary material for: Genomic analysis of the relationship between gene expression variation and DNA polymorphism in Drosophila simulans
Source: Genome Biol. 2008 Aug 12;9(8):R125. doi: 10.1186/gb-2008-9-8-r125 (PMC2575515; doi:10.1186/gb-2008-9-8-r125)
Supplement: Additional data file 2 — Details of the statistical results summarized in Table 4. [file gb-2008-9-8-r125-S2.doc]

Table S2

| **Gene feature length, polymorphism and divergence for sex-specific, sex-biased, and unbiased genes.** | | | | | | | | | | | | | | |
| --- | --- | --- | --- | --- | --- | --- | --- | --- | --- | --- | --- | --- | --- | --- |
|  | Genome Average |  | Male^a^ | |  | Unbiased |  | Female^a^ | |  | *X^2^* | *p*-value^b^ | Tukey's HSD summary^c^ | Summary^d^ |
|  |  |  | Specific | Biased |  |  |  | Specific | Biased |  |  |  |  |  |
| Num. Genes | 8471 |  | 1343 | 150 |  | 5246 |  | 522 | 1210 |  |  |  |  |  |
|  |  |  |  |  |  |  |  |  |  |  |  |  |  |  |
| **Length** |  |  |  |  |  |  |  |  |  |  |  |  |  |  |
| EXON | 1675 |  | 1370 | 1302 |  | 1681 |  | 1785 | 1986 |  | 247.10 | *** | Fb ≥ Fs ≥ U ≥ Mb ≥ Ms | F>U>M |
| 5'UTR | 239 |  | 197 | 212 |  | 252 |  | 180 | 241 |  | 133.27 | *** | U,Fb ≥ Mb ≥ Ms,Fs | NS>SS |
| Intron | 2493 |  | 1920 | 2867 |  | 2785 |  | 1045 | 2321 |  | 131.81 | *** | U ≥ Mb,Fb,Ms > Fs | NS>SS |
| Num. Introns | 3.55 |  | 3.15 | 2.94 |  | 3.70 |  | 2.89 | 3.62 |  | 64.44 | *** | U,Fb ≥ Mb ≥ Ms,Fs | NS>SS |
| 3'UTR | 392 |  | 278 | 300 |  | 423 |  | 273 | 402 |  | 236.01 | *** | U ≥ Fb ≥ Mb > Ms,Fs | NS>SS |
| 5’ intergenic | 3377 |  | 6579 | 3709 |  | 3085 |  | 2321 | 1942 |  | 291.9 | *** | Ms > Mb,U, Fs ≥ Fb | M>F, U |
| 3’ intergenic | 2619 |  | 5378 | 3523 |  | 2236 |  | 1966 | 1760 |  | 274.6 | *** | Ms ≥ Mb ≥ U, Fb, Fs | M>F, U |
|  |  |  |  |  |  |  |  |  |  |  |  |  |  |  |
| **Polymorphism** |  |  |  |  |  |  |  |  |  |  |  |  |  |  |
| CPR | 0.0290 |  | 0.0233 | 0.0215 |  | 0.0296 |  | 0.0299 | 0.0313 |  | 79.64 | *** | Fb,Fs,U > Ms,Mb | F,U>M |
| 5'UTR | 0.0112 |  | 0.0133 | 0.0105 |  | 0.0106 |  | 0.0128 | 0.0118 |  | 22.14 | 0.0002 | Ms,Fs ≥ Fb,Mb ≥ U | SS>NS |
| Nonsynon. | 0.0024 |  | 0.0037 | 0.0027 |  | 0.0020 |  | 0.0033 | 0.0020 |  | 305.11 | *** | Ms ≥ Fs ≥ Mb ≥ U,Fb | SS>NS |
| Synon. | 0.0318 |  | 0.0337 | 0.0330 |  | 0.0313 |  | 0.0350 | 0.0305 |  | 33.62 | *** | Fs,Ms ≥ Mb ≥ U,Fb | SS>NS |
| first Intron | 0.0277 |  | 0.0344 | 0.0316 |  | 0.0267 |  | 0.0287 | 0.0259 |  | 59.49 | *** | Ms ≥ Mb ≥ Fs,U,Fb | M>F,U |
| all Intron | 0.0302 |  | 0.0365 | 0.0350 |  | 0.0290 |  | 0.0306 | 0.0292 |  | 48.10 | *** | Ms ≥ Mb ≥ Fs,Fb,U | M>F,U |
| 3'UTR | 0.0122 |  | 0.0185 | 0.0155 |  | 0.0114 |  | 0.0149 | 0.0099 |  | 156.48 | *** | Ms ≥ Mb ≥ Fs > U >Fb | M>F,U |
|  |  |  |  |  |  |  |  |  |  |  |  |  |  |  |
| **Divergence**^e^ |  |  |  |  |  |  |  |  |  |  |  |  |  |  |
| CPR | 0.0525 |  | 0.0419 | 0.0373 |  | 0.0522 |  | 0.0578 | 0.0620 |  | 212.79 | *** | Fb,Fs > U > Ms,Mb | F>U>M |
| 5'UTR | 0.0229 |  | 0.0280 | 0.0224 |  | 0.0210 |  | 0.0301 | 0.0245 |  | 80.02 | *** | Fs ≥ Ms > Fb ≥ Mb ≥ U | SS>NS |
| Nonsynon. | 0.0060 |  | 0.0114 | 0.0072 |  | 0.0046 |  | 0.0076 | 0.0051 |  | 533.92 | *** | Ms > Fs,Mb > Fb,U | SS>NS |
| Synon. | 0.0531 |  | 0.0566 | 0.0516 |  | 0.0516 |  | 0.0575 | 0.0536 |  | 81.82 | *** | Ms ≥ Fs,Fb,Mb ≥ U | SS>NS |
| first Intron | 0.0463 |  | 0.0552 | 0.0492 |  | 0.0443 |  | 0.0523 | 0.0456 |  | 68.47 | *** | Ms,Fs ≥ Mb ≥ Fb,U | SS>NS |
| all Intron | 0.0487 |  | 0.0574 | 0.0462 |  | 0.0468 |  | 0.0574 | 0.0485 |  | 55.72 | *** | Ms ≥ Fs ≥ Mb ≥ Fb,U | SS>NS |
| 3'UTR | 0.0228 |  | 0.0349 | 0.0273 |  | 0.0202 |  | 0.0325 | 0.0210 |  | 259.87 | *** | Ms ≥ Fs ≥ Mb ≥ Fb ≥ U | SS>NS |

^a^ Male- and female-specific sets include genes that are expressed only in that sex, whereas sex-biased are expressed on average 3-fold higher in one sex than the other.

^b^ *X^2^* and *p*-values derived from Kruskal Wallis; *** denotes *p*-value <0.0001.

^c^ Ms = male-specific, Mb = male-biased, Fs = female-specific, Fb = female-biased, U = unbiased.

^d^ F = female, M = male, U = unbiased, NS = not sex-specific, SS = sex-specific.

^e^ Divergence refers to lineage specific divergence along the *D. simulans* branch.
