# Supplementary material for: Genomic analysis of the relationship between gene expression variation and DNA polymorphism in Drosophila simulans
Source: Genome Biol. 2008 Aug 12;9(8):R125. doi: 10.1186/gb-2008-9-8-r125 (PMC2575515; doi:10.1186/gb-2008-9-8-r125)
Supplement: Additional data file 3 — The relationship between expression variation and divergence for the gene features discussed in this manuscript. [file gb-2008-9-8-r125-S3.doc]

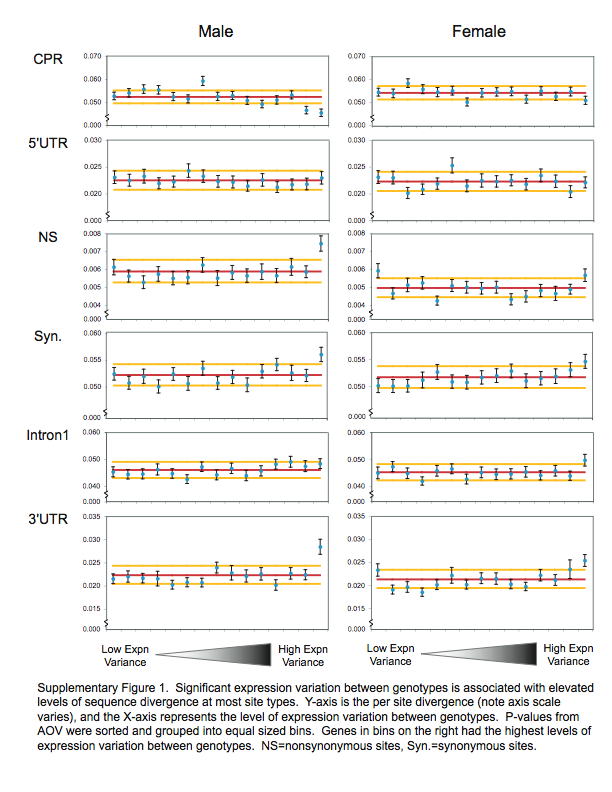


# NonSyn

# Syn

# Figure S1

Significant expression variation between genotypes is associated with elevated levels of sequence divergence at most site types. Y-axis is the per site divergence (note axis scale varies), and the X-axis represents the level of expression variation between genotypes. P-values from AOV were sorted and grouped into equal sized bins. Genes in bins on the right had the highest levels of expression variation between genotypes. NonSyn = nonsynonymous sites; Syn = synonymous sites.
